# Incidence and Prevalence of Post-COVID-19 Myalgic Encephalomyelitis: A Report from the Observational RECOVER-Adult Study

**DOI:** 10.1007/s11606-024-09290-9

**Published:** 2025-01-13

**Authors:** Suzanne D. Vernon, Tianyu Zheng, Hyungrok Do, Vincent C. Marconi, Leonard A. Jason, Nora G. Singer, Benjamin H. Natelson, Zaki A. Sherif, Hector Fabio Bonilla, Emily Taylor, Janet M. Mullington, Hassan Ashktorab, Adeyinka O. Laiyemo, Hassan Brim, Thomas F. Patterson, Teresa T. Akintonwa, Anisha Sekar, Michael J. Peluso, Nikita Maniar, Lucinda Bateman, Leora I. Horwitz, Rachel Hess, on behalf of the NIH Researching COVID to Enhance Recovery (RECOVER) Consortium

**Affiliations:** 1 Bateman Horne Center, 24 S 1100 E Suite 205, Salt Lake City, UT USA; 2 Department of Population Health Sciences, Spencer Fox Eccles School of Medicine at the University of Utah, Salt Lake City, UT USA; 3 Department of Population Health at NYU Grossman School of Medicine, New York University, New York, NY USA; 4 Rollins School of Public Health, Atlanta Veterans Affairs Medical Center, and the Emory Vaccine Center, Emory University School of Medicine, Atlanta, GA USA; 5 Center for Community Research, DePaul University, Chicago, IL USA; 6 Division of Rheumatology, The MetroHealth System, Case Western Reserve University, Cleveland, OH USA; 7 Pain and Fatigue Study Center, Department of Neurology, Icahn School of Medicine at Mount Sinai, New York, NY USA; 8 Department of Biochemistry & Molecular Biology, District of Columbia, Howard University College of Medicine, Washington, USA; 9 Department of Medicine, Division of Infectious Diseases, Stanford University, Palo Alto, CA USA; 10 Solve ME/CFS Initiative, Glendale, CA USA; 11 Department of Neurology, Beth Israel Deaconess Medical Center, Harvard Medical School, Boston, MA USA; 12 Department of Medicine, Division of Gastroenterology, Howard University College of Medicine, Washington, District of Columbia USA; 13 Department of Pathology, Howard University College of Medicine, Washington, District of Columbia USA; 14 Division of Infectious Diseases, The University of Texas Health Science Center at San Antonio, San Antonio, TX USA; 15 Black COVID Survivors Alliance, Inc, Atlanta, GA USA; 16 Patient-Led Research Collaborative, San Francisco, CA USA; 17 Division of HIV, Infectious Diseases, and Global Medicine, University of California, San Francisco, CA USA; 18 Department of Neurology & Rehabilitation, University of Illinois at Chicago, Chicago, IL USA; 19 Division of Healthcare Delivery Science, Department of Population Health, NYU Grossman School of Medicine, New York University, New York, NY USA; 20 Department of Internal Medicine, Spencer Fox Eccles School of Medicine at the University of Utah, Salt Lake City, UT USA

**Keywords:** Post-COVID-19 ME/CFS, ME/CFS, SARS-CoV-2, RECOVER

## Abstract

**Background:**

Myalgic encephalomyelitis/chronic fatigue syndrome (ME/CFS) may occur after infection. How often people develop ME/CFS after SARS-CoV-2 infection is unknown.

**Objective:**

To determine the incidence and prevalence of post-COVID-19 ME/CFS among adults enrolled in the Researching COVID to Enhance Recovery (RECOVER-Adult) study.

**Design, Setting, and Participants:**

RECOVER-Adult is a longitudinal observational cohort study conducted across the U.S. We included participants who had a study visit at least 6 months after infection and had no pre-existing ME/CFS, grouped as (1) acute infected, enrolled within 30 days of infection or enrolled as uninfected who became infected (*n*=4515); (2) post-acute infected, enrolled greater than 30 days after infection (*n*=7270); and (3) uninfected (1439).

**Measurements:**

Incidence rate and prevalence of post-COVID-19 ME/CFS based on the 2015 Institute of Medicine ME/CFS clinical diagnostic criteria.

**Results:**

The incidence rate of ME/CFS in participants followed from time of SARS-CoV-2 infection was 2.66 (95% CI 2.63–2.70) per 100 person-years while the rate in matched uninfected participants was 0.93 (95% CI 0.91–10.95) per 100 person-years: a hazard ratio of 4.93 (95% CI 3.62–6.71). The proportion of all RECOVER-Adult participants that met criteria for ME/CFS following SARS-CoV-2 infection was 4.5% (531 of 11,785) compared to 0.6% (9 of 1439) in uninfected participants. Post-exertional malaise was the most common ME/CFS symptom in infected participants (24.0%, 2830 of 11,785). Most participants with post-COVID-19 ME/CFS also met RECOVER criteria for long COVID (88.7%, 471 of 531).

**Limitations:**

The ME/CFS clinical diagnostic criteria uses self-reported symptoms. Symptoms can wax and wane.

**Conclusion:**

ME/CFS is a diagnosable sequela that develops at an increased rate following SARS-CoV-2 infection. RECOVER provides an unprecedented opportunity to study post-COVID-19 ME/CFS.

**Supplementary Information:**

The online version contains supplementary material available at 10.1007/s11606-024-09290-9.

## INTRODUCTION

Myalgic encephalomyelitis/chronic fatigue syndrome (ME/CFS) can be triggered following acute infection. A landmark prospective study followed patients from the time of acute infection with Epstein-Barr virus, *Coxiella burnetii*, or Ross River virus and found that 11% met ME/CFS criteria at 6 months post-infection.^1^

Researching COVID to Enhance Recovery (RECOVER) is a national initiative funded by the National Institutes of Health to conduct research on post-COVD-19 conditions, including postacute sequelae of SARS-CoV-2 infection (PASC), also known as long COVID.^2^ One component of RECOVER, the longitudinal observational adult cohort (RECOVER-Adult), reported that 85% of participants with PASC had fatigue.^3^ Other symptoms that PASC patients experience include post-exertional malaise (PEM), brain fog, dizziness, and unrefreshing sleep. These are consistent with the core symptoms that are diagnostic for ME/CFS that include new onset of fatigue that has persisted for at least 6 months and is accompanied by a reduction in pre-illness activities, post-exertional malaise (PEM), and unrefreshing sleep plus either cognitive impairment or orthostatic intolerance (OI).^4^

We applied the ME/CFS clinical diagnostic criteria to (1) determine the incidence rate of post-COVID-19 ME/CFS among prospectively followed participants enrolled within 30 days of SARS-CoV-2 infection, and (2) compare the occurrence of new onset ME/CFS in participants with and without SARS-CoV-2 infection who were enrolled in the RECOVER-Adult study. We hypothesized that there would be an increased rate of ME/CFS in RECOVER-Adult participants infected with SARS-CoV-2 compared to matched uninfected participants.

## METHODS

The study was approved by the institutional review board at NYU Grossman School of Medicine, serving as a single institutional review board. All participants provided written informed consent. The study is registered at NCT05172024.

### Study Design

RECOVER-Adult includes participants with and without SARS-CoV-2 infection.^2^ Participants with SARS-CoV-2 infection met World Health Organization suspected, probable, or confirmed criteria for infection and were enrolled in RECOVER from 83 sites in 33 states plus Puerto Rico and Washington, DC.^5^ Uninfected individuals did not meet any WHO criteria for infection and had a documented negative SARS-CoV-2 nucleic acid and nucleocapsid antibody test result at the time of enrollment.

### Participants

The RECOVER-Adult study included 15,181 people enrolled as (1) acute infected, enrolled within 30 days of infection; (2) post-acute infected, enrolled greater than 30 days after infection; or (3) uninfected. As SAR-CoV-2 infections continued to occur in study participants, participants who were initially enrolled as uninfected and then became infected during the study were reclassified as acute infected for post-infection visits (*n*=954) and were not included in the uninfected analyses. We excluded participants people who were hospitalized for COVID-19 (*n*=294), those who did not respond to the symptom questionnaires (*n*=640), any infected participant that did not undergo a study visit ≥6-month after incident SARS-CoV-2 infection (*n*=773), and those with pre-existing ME/CFS (*n*=198 infected, *n*=22 uninfected). The remaining 11,785 infected participants were assigned to three groups using the first qualifying visit at least 6 months from index infection: (1) post-COVID-19 ME/CFS participants, (2) ME/CFS-like participants, and (3) participants who did not report any ME/CFS symptoms.

The 1439 uninfected participants were classified to parallel the infected participant groups.

### Exposure

The exposure was SARS-CoV-2 infection.

### Data Source

Outcome measures were drawn from self-reported symptoms and comorbid medical conditions, as reported by participants at 3-month interval study visits.^2^ All data were ascertained from the September 2024 data lock.

### Outcome

The primary outcome was new ME/CFS determined using the Institute of Medicine (IOM) ME/CFS clinical diagnostic criteria that require fatigue accompanied by physical impairment, PEM, unrefreshing sleep and either cognitive impairment or OI.^4^ The first symptom survey response at least 6 months, or later from first infection was used to group infected participants as post-COVID-19 ME/CFS and ME/CFS-like; for uninfected participants, we included symptoms from any survey response.

### Questionnaires to Assess ME/CFS Symptoms

Patient-Reported Outcomes Measurement Information System (PROMIS) Global Health 10 was used to assess (1) severity of fatigue over the past 7 days and (2) physical impairment by inability to carry out daily physical activities. PROMIS Sleep Disturbance question “My sleep was refreshing” was used to assess the severity of unrefreshing sleep over the past 7 days; responses of “Not at all” or “A little bit” were considered moderate to severe unrefreshing sleep. Cognitive impairment was determined by having a self-reported Neuro-QoL cognition T-score of ≤40 (1 SD below national mean) or a raw Neuro-QoL score of <24.^3,6^ PEM and OI were assessed with a question asking if the participant had “post-exertional malaise (symptoms worse after even minor physical or mental effort)” (PEM) and “Feeling faint, dizzy, ‘goofy’; difficulty thinking soon after standing up from a sitting or lying position” (OI), respectively. Responses that indicated the presence of PEM or OI included “Yes, I have it NOW,” or “Yes, and I STILL HAVE IT.” Frequency and severity measures were not available for these symptoms; thus, all positive symptoms were considered qualifying.

ME/CFS was defined as reporting moderate to very severe fatigue over the past 7 days plus moderate to complete interference with ability to carry out every day physical activities, AND presence of PEM, AND not at all or a little bit of refreshing sleep over the past 7 days PLUS the presence of OI and/or a Neuro-QoL cognitive T-score of <40 or raw score of <24. Those having at least one ME/CFS symptom but not meeting all the above criteria were considered ME/CFS-like.

The RECOVER case definition for PASC identified four clusters composed of 44 symptoms among those with PASC.^3^ Cluster 1 was characterized primarily by loss/change in smell or taste, and those participants had lowest symptom burden and quality of life impairment. Nearly all participants in cluster 2 had PEM (99%); half had dizziness and gastrointestinal symptoms; none had brain fog. Cluster 3 was defined by the presence of brain fog (100%) in addition to PEM (99%) and other symptoms. Cluster 4 participants had high levels of all symptoms, including PEM, dizziness, gastrointestinal symptoms, and brain fog, and overall had the worst quality of life. We determined which cluster post-COVID-19 ME/CFS and ME/CFS-like participants were assigned to in the study visit used for analysis.

### Statistical Analysis

RECOVER-Adult data collected between October 2021 and September 2024, and stored on the RECOVER analytic platform, Seven Bridges, were used in this analysis. Analytic groups were created using the ME/CFS criteria. Chi-square tests were used to assess differences in demographics between analytic groups and the two-sample *t*-test to test whether age at enrollment differed among the infected and uninfected groups. Fisher’s exact test was used to determine differences in comorbid conditions between infected and uninfected participants. All analyses were conducted by restricting infected participants to those enrolled within 30 days of the first infection and then repeated the analyses with all participants regardless of time from SARS-CoV2 infection.

The incidence rate of post-COVID-19 ME/CFS was calculated among participants enrolled <30 days after acute SARS-CoV-2 infection compared to matched uninfected controls. We used propensity score matching with replacement to minimize selection bias given differences in baseline characteristics between infected and uninfected participants.^7^ Propensity scores were estimated using logistic regression and adjusting for potential demographic and comorbidity condition confounders that were unrelated to SARS-CoV-2 infection but had features that were related to ME/CFS (Supplemental Material Table 1). We paired each uninfected participant with the acutely infected participant who had the closest propensity score that was within 0.2 standard deviations of the logit of propensity score (“greedy matching”). Among the 1439 uninfected participants, 847 (58.9%) had no matched acute infected participants, indicating substantial differences between the characteristics of the acutely infected and uninfected cohorts. After matching, the standard mean differences (SMD) were 0.036 meaning that the propensity score matching was successful. The demographic characteristics and comorbid conditions in the acute infected and uninfected groups before and after propensity matching are provided (Supplemental Material Tables 2 and 3). The hazard ratio for acute COVID-19 infection compared with no infection was calculated. Finally, we determined which PASC cluster the post-COVID-19 ME/CFS and ME/CFS-like participants were assigned to at their first qualifying visit. We considered a two-sided *P* value of less than 0.05 to be statistically significant. Analyses were conducted using SAS Studio, R, and Python 3.11 with scikit-learn (1.3.1; http://scikit-learn.org), lifelines (0.29.1; https://github.com/CamDavidsonPilon/lifelines), and pymatch (0.3.4; https://github.com/benmiroglio/pymatch) packages.

**Table 1:** Demographic Characteristics of *Infected* Participants of the RECOVER-Adult Study

	Infected groups (number (%))			*P* value	
	Post-COVID-19 ME/CFS	ME/CFS-like	Never met criteria	Post-COVID-19 ME/CFS vs never	ME/CFS-like vs never
Enrollment age				<0.001	0.076
Median (IQR)	48 (18)	45 (24)	43 (26)		
*N*	531	4692	6562		
Age category at enrollment (*n*, %)				<0.001	<0.001
18–45	223 (42.0)	2367 (50.5)	3512 (53.5)		
46–65	275 (51.8)	1757 (37.5)	2080 (31.7)		
>65	33 (6.1)	566 (12.1)	968 (14.8)		
Missing (<18)	0 (0)	2 (0.04)	2 (0.03)		
Sex at birth				<0.001	<0.001
Female	422 (79.5)	3552 (75.7)	4626 (70.5)		
Intersex	0 (0)	1 (0.02)	3 (0.05)		
Male	107 (20.2)	1114 (23.8)	1913 (29.2)		
Missing	2 (0.4)	25 (0.5)	20 (0.3)		
Race				<0.001	<0.001
Asian, non-Hispanic	15 (2.8)	211 (4.5)	464 (7.1)		
Black, non-Hispanic	45 (8.5)	630 (13.4)	941 (14.3)		
Hispanic	77 (14.5)	673 (14.3)	871 (13.3)		
Multiracial/ethnic	49 (9.2)	402 (8.6)	457 (7.0)		
White, non-Hispanic	328 (61.8)	2646 (56.4)	3661 (55.8)		
Other	15 (28)	106 (2.3)	144 (2.2)		
Missing	2 (0.4)	24 (0.5)	24 (0.4)		
Vaccine at enrollment				<0.001	0.068
Yes	459 (86.4)	4203 (89.6)	5945 (90.6)		
No	58 (10.9)	356 (7.6)	440 (6.7)		
Don’t know	0 (0)	2 (0.04)	4 (0.1)		
Missing	11 (2.1)	107 (2.3)	150 (2.3)		
Prefer not to answer	3 (0.6)	24 (0.5)	23 (0.4)		
Education				<0.001	<0.001
Bachelors/advanced degree	291 (54.8)	2832 (60.4)	4602 (70.1)		
High school/some college	217 (40.9)	1659 (35.4)	1716 (26.2)		
Not complete high school	15 (2.8)	151 (3.2)	186 (2.8)		
Missing	3 (0.6)	30 (0.6)	35 (0.5)		
Prefer not to answer	5 (1.0)	20 (0.4)	23 (0.4)		
Rural				<0.001	<0.001
No	493 (92.8)	4421 (94.2)	6324 (96.4)		
Yes	38 (7.2)	271 (5.8)	238 (3.6)		
Medically underserved area				0.616	<0.001
No	407 (76.7)	3384 (72.1)	4966 (75.7)		
Yes	124 (23.4)	1308 (27.9)	1596 (24.3)

**Table 2:** Demographic Characteristics of *Uninfected* Participants of the RECOVER-Adult Study

	Uninfected groups (number (%))			*P* value	
	ME/CFS	ME/CFS-like	Never met criteria	ME/CFS vs never	ME/CFS-like vs never
Enrollment age				0.758	0.653
Median (IQR)	44 (21)	51 (27)	49 (27)		
*N*	9	232	1198		
Age category at enrollment (*n*, %)				0.826	0.882
18–45	5 (55.6)	104 (44.8)	554 (46.2)		
46–65	3 (33.3)	89 (38.4)	439 (36.6)		
>65	1 (11.1)	39 (16.8)	205 (17.1)		
Missing (<18)	0 (0)	0 (0)	0 (0)		
Sex at birth				<0.001	0.258
Female	4 (44.4)	145 (62.5)	815 (68.0)		
Male	4 (44.4)	86 (37.1)	378 (31.6)		
Missing	1 (1.1)	1 (0.4)	5 (0.4)		
Race				0.589	0.911
Asian, non-Hispanic	0 (0)	15 (6.5)	75 (6.3)		
Black, non-Hispanic	1 (11.1)	46 (19.8)	238 (19.8)		
Hispanic	0 (0)	24 (10.3)	109 (9.1)		
Multiracial/ethnic	0 (0)	21 (19.1)	88 (7.4)		
White, non-Hispanic	7 (77.8)	121(52.2)	657 (54.8)		
Other	0 (0)	4 (1.7)	26 (2.2)		
Missing	1 (1.1)	1 (0.4)	5 (0.4)		
Vaccine at enrollment				0.550	0.654
Yes	7 (77.8)	208 (89.7)	1,098 (91.7)		
No	0 (0)	9 (3.9)	56 (4.7)		
Don’t know	0 (0)	1 (0.4)	0 (0)		
Missing	2 (22.2)	13 (5.6)	43 (3.6)		
Prefer not to answer	0 (0)	1 (0.4)	1 (0.1)		
Education				0.726	<0.001
Bachelors/advanced degree	4 (44.4)	123 (53.0)	772 (64.4)		
High school/some college	3 (33.3)	88 (37.9)	372 (31.2)		
Not complete high school	0 (0)	19 (8.2)	47 (3.9)		
Missing	2 (22.2)	1 (0.4)	6 (0.5)		
Prefer not to answer	0 (0)	1 (0.4)	1 (0.1)		
Rural				0.515	0.203
No	9 (100.0)	217 (93.5)	1144 (95.5)		
Yes	0 (0)	15 (6.5)	54 (4.5)		
Medically underserved area				0.068	0.138
No	9 (100.0)	158 (68.1)	873 (732.9)		
Yes	0 (0)	74 (31.9)	325 (27.1)

**Table 3:** Comorbidities in Post-COVID-19 ME/CFS and ME/CFS-like Compared to those Who Never Met ME/CFS Criteria and Total Infected and Uninfected

	Post-COVID-19 ME/CFS (*n*=531)	ME/CFS-like (*n*=4692)	Never Met Criteria (*n*=6562)	*P* value	Infected (*n*=11,785)	Uninfected (*n*=1439)	*P* value
Anxiety, depression or PTSD	68 (12.8%)	891 (19%)	908 (13.8%)	<.0001	1867 (15.8%)	531 (36.9%)	<.0001
Cardiovascular disease	43 (8.1%)	492 (10.5%)	774 (11.8%)	.0063	1309 (11.1%)	389 (27%)	<.0001
Obesity	52 (9.8%)	659 (14%)	831 (12.7%)	.0064	1542 (13.1%)	351 (24.4%)	<.0001
Asthma	49 (9.2%)	429 (9.1%)	544 (8.3%)	.2428	1022 (8.7%)	237 (16.5%)	<.0001
Other mental health disorder	21 (4%)	215 (4.6%)	175 (2.7%)	<.0001	411 (3.5%)	233 (16.2%)	<.0001
Rheumatologic, autoimmune or connective tissue disease	32 (6%)	262 (5.6%)	309 (4.7%)	.0697	603 (5.1%)	161 (11.2%)	<.0001
Diabetes and specific type	17 (3.2%)	210 (4.5%)	306 (4.7%)	.2932	533 (4.5%)	140 (9.7%)	<.0001
Immunocompromised condition	23 (4.3%)	139 (3%)	185 (2.8%)	.1434	347 (2.9%)	143 (9.9%)	<.0001
Chronic pain syndrome or fibromyalgia	37 (7%)	169 (3.6%)	76 (1.2%)	<.0001	282 (2.4%)	102 (7.1%)	<.0001
Neuromuscular disease	16 (3.0%)	125 (2.7%)	79 (1.2%)	<.0001	220 (1.9%)	85 (5.9%)	<.0001
Other chronic lung disease	10 (1.9%)	58 (1.2%)	61 (0.9%)	.0592	129 (1.1%)	67 (4.7%)	<.0001
Active cancer	5 (0.9%)	61 (1.3%)	116 (1.8%)	.0823	182 (1.5%)	61 (4.2%)	<.0001
Schizophrenia or bipolar disorder	9 (1.7%)	75 (1.6%)	61 (0.9%)	.0032	145 (1.2%)	82 (5.7%)	<.0001
Chronic obstructive pulmonary disease	14 (2.6%)	77 (1.6%)	56 (0.9%)	<.0001	147 (1.3%)	62 (4.3%)	<.0001
Kidney disease	4 (0.8%)	84 (1.8%)	92 (1.4%)	.0875	180 (1.5%)	53 (3.7%)	<.0001
Polycystic ovary disease	5 (0.9%)	99 (2.1%)	126 (1.9%)	.1675	230 (2%)	54 (3.8%)	<.0001
Chronic liver disease	6 (1.1%)	38 (0.8%)	51 (0.8%)	.6118	95 (0.8%)	38 (2.6%)	<.0001
Stroke or bleed	9 (1.7%)	40 (0.9%)	55 (0.8%)	.1337	104 (0.9%)	38 (2.6%)	<.0001
Dementia or cognitive impairment	9 (1.7%)	62 (1.3%)	35 (0.5%)	<.0001	106 (0.9%)	32 (2.2%)	<.0001
Movement disorder	6 (1.1%)	38 (0.8%)	25 (0.4%)	.0021	69 (0.6%)	29 (2.0%)	<.0001
POTS, dysautonomia or autonomic dysfunction	9 (1.7%)	45 (1.0%)	21 (0.3%)	<.0001	75 (0.6%)	25 (1.7%)	<.0001
Seizure disorder	5 (0.9%)	38 (0.8%)	34 (0.5%)	.0908	77 (0.7%)	16 (1.1%)	.0635
Use of oxygen at home	9 (1.7%)	22 (0.5%)	23 (0.4%)	.0011	54 (0.5%)	22 (1.5%)	<.0001
Sickle cell anemia	2 (0.4%)	9 (0.2%)	7 (0.1%)	.1411	18 (0.2%)	16 (1.1%)	<.0001
CNS infection, inflammatory or demyelinating disease	3 (0.6%)	23 (0.5%)	18 (0.3%)	.0974	44 (0.4%)	10 (0.7%)	.0787

## RESULTS

**Figure 1:**
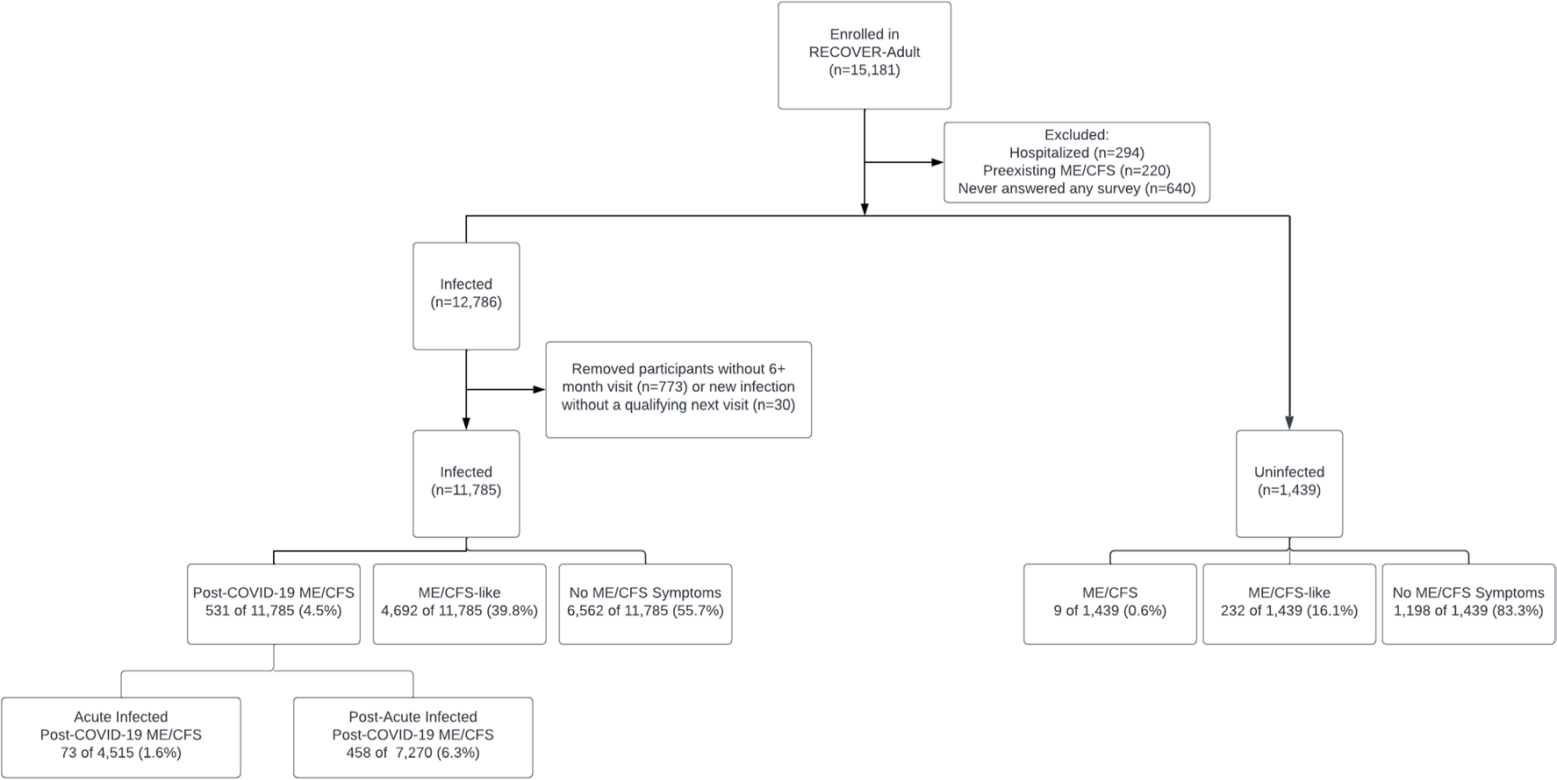
The ME/CFS clinical diagnostic criteria was applied to both infected and uninfected participants in the RECOVER Adult study group

ME/CFS prevalence was determined among infected participants and uninfected participants (Fig. 1). Among the 11,785 infected participants, 531 (4.5%) met ME/CFS diagnostic criteria at the first study visit at least 6 months after acute SARS-CoV-2 infection; 4692 (39.8%) were ME/CFS-like, with at least one ME/CFS symptom; and 6562 (55.7%) did not report any ME/CFS symptoms. Of the 1439 uninfected participants, 9 (0.6%) met ME/CFS clinical diagnostic criteria, 232 (16.1%) had at least one ME/CFS symptom, and 1198 (83.3%) did not report any ME/CFS symptoms.

The 4515 acute infected participants provided the opportunity to estimate the incidence of new post-COVID-19 ME/CFS cases. The incidence rate of ME/CFS among acute infected participants was 2.66 (95% CI 2.63–2.70) per 100 person-years, which was significantly greater than the incidence rate in the propensity score-matched uninfected participants (0.93 per 100 person-years, 95% CI 0.91–0.95) (*p*<.0001). This is an attributable risk of 1.74 per 100 person-years with a significantly different incidence rate ratio test between SARS-CoV-2 infected and uninfected participants (*p* <.0001).^8^ Furthermore, the hazard ratio for developing ME/CFS in acute infected participants compared to uninfected participants was 4.93 (95% CI 3.62–6.71) (*p*<.005).

Compared to those never meeting ME/CFS criteria in the infected cohort, those with post-COVID-19 ME/CFS were more likely to be White, female, between 46 to 65 years of age, and live in a rural area, and less likely to have been vaccinated at enrollment and to have completed college (Table 1). Since there were only nine participants that met ME/CFS criteria in the uninfected cohort, the sample size was too small to compare to the other uninfected groups (Table 2).

Post-COVID-19 ME/CFS participants were more likely to report chronic pain syndrome or fibromyalgia, neuromuscular disease, chronic obstructive pulmonary disease, dementia or cognitive impairment, postural orthostatic tachycardia syndrome (POTS), dysautonomia or autonomic dysfunction, movement disorder, other mental health disorder, and use of oxygen at home compared to uninfected participants (Table 3). The remaining medical and psychiatric conditions occurred at similar or lower rates compared to those who did not meet ME/CFS criteria.

**Figure 2:**
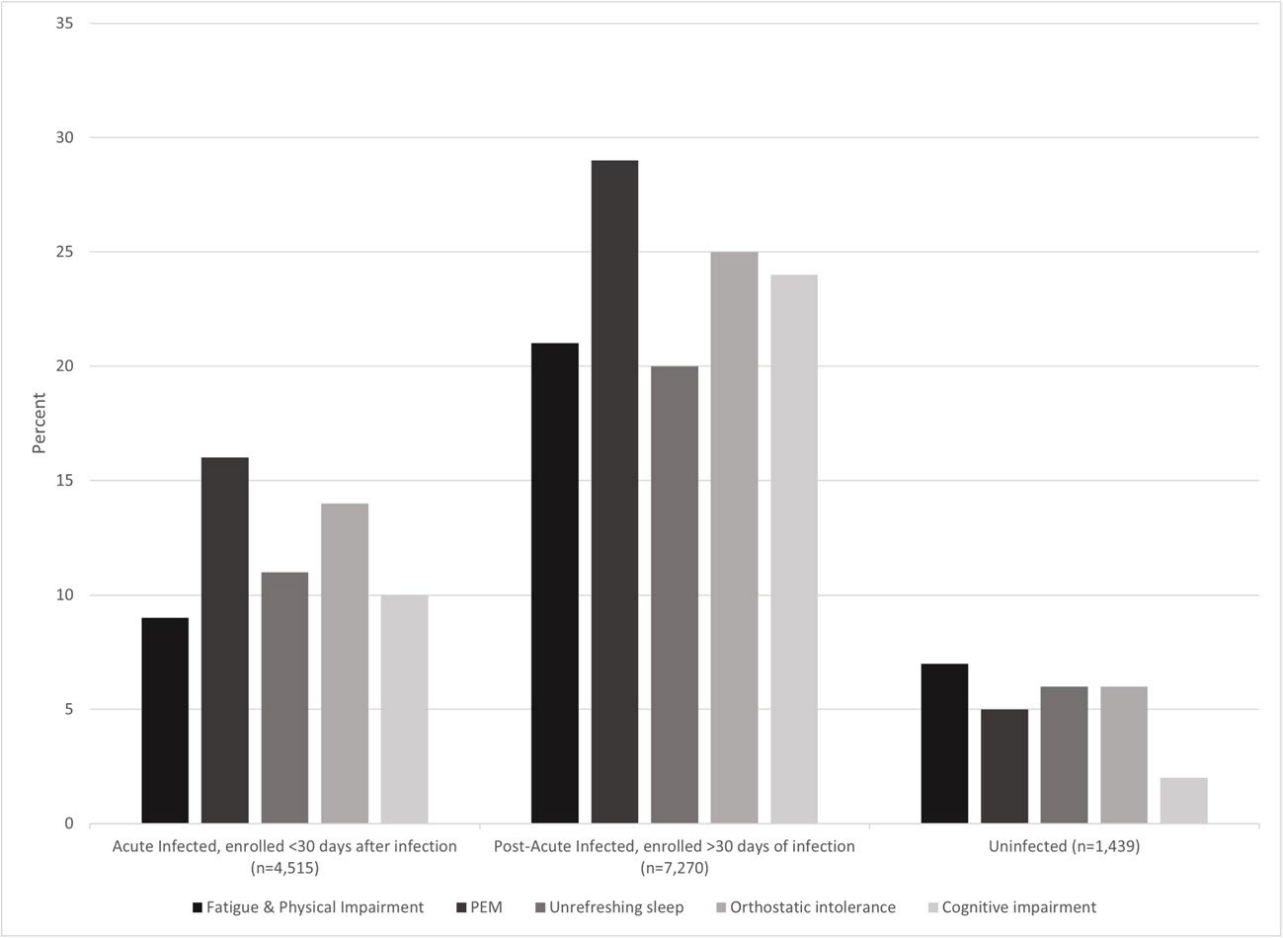
Percent of infected and uninfected participants with ME/CFS symptoms

We assessed the proportion of acute infected, post-acute infected, and uninfected participants who reported each ME/CFS symptom (Fig. 2). PEM was the most frequently reported symptom in both acute infected participants (15.9%, 717/4515) and post-acute infected participants (29.1%, 2113/7270). OI was the next most common symptom, reported in 14.4% (652/4515) of acute infected participants and in 25.0% (1815/7270) of post-acute infected participants. Unrefreshing sleep (11.0%, 498/4515), cognitive impairment (10.1%, 457/4515), and fatigue (9.3%, 418/4515) were reported at similar rates in acute infected participants. Cognitive impairment occurred in 23.7% (1725/7270) of post-acute infected participants followed by fatigue (20.7%, 1508/7270) then unrefreshing sleep (19.8%, 1436/7270). All ME/CFS symptoms were lower in uninfected compared to infected participants.

**Figure 3:**
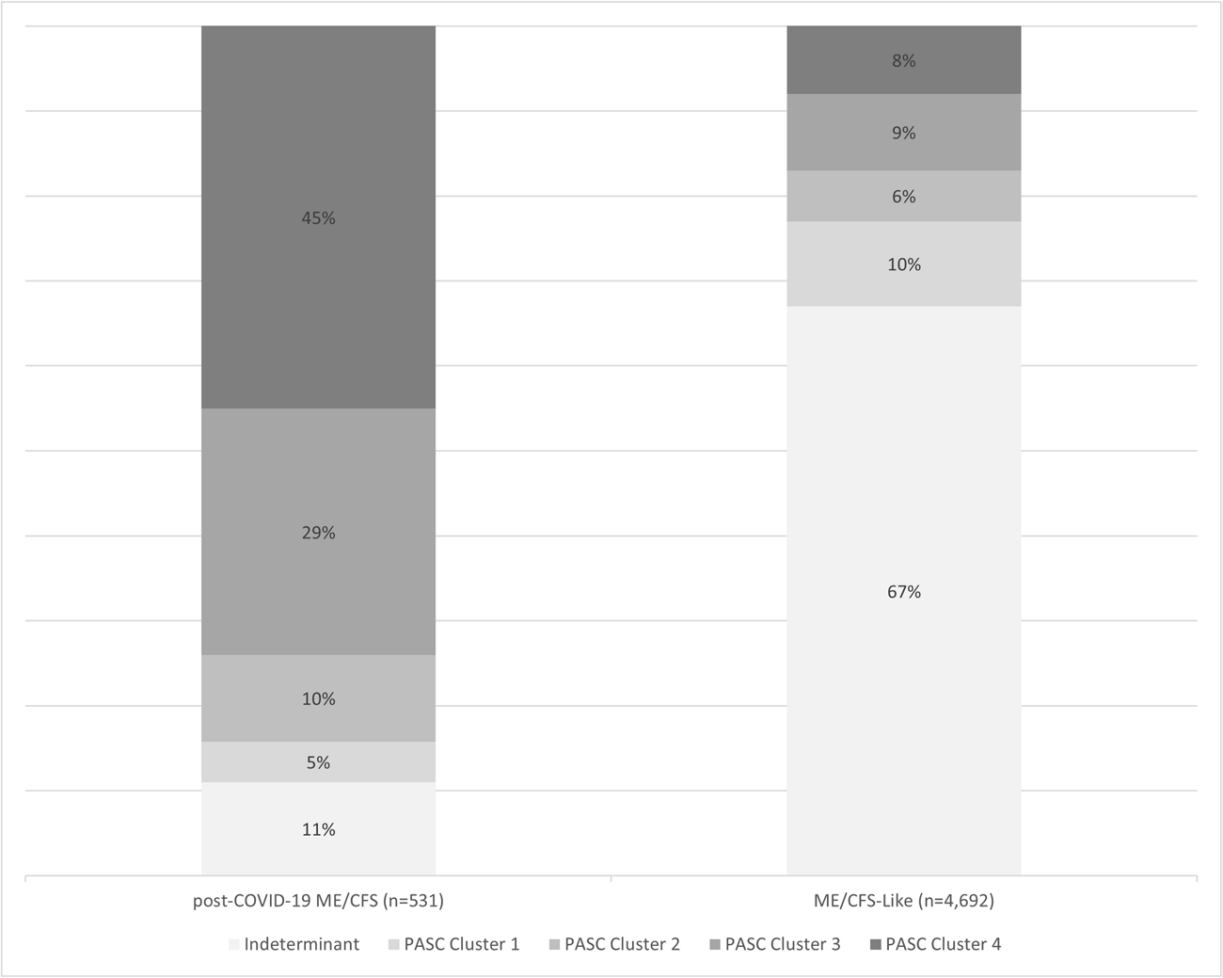
PASC cluster assignments at the first qualifying visit for post-COVID-19 ME/CFS and ME/CFS-like participants

Eighty-nine percent (471/531) of post-COVID-19 ME/CFS participants met PASC criteria and most (45.0%, 239/531) were assigned to PASC cluster 4, the cluster with the highest frequency of all symptoms including ME/CFS symptoms (Fig. 3). Twenty-nine percent (156/531) of post-COVID-19 ME/CFS participants were assigned to cluster 3, 10% (52/531) were assigned to cluster 2, and 5% (24/531) were assigned to cluster 1. Eleven percent of post-COVID-19 ME/CFS participants were PASC indeterminant. In contrast, most ME/CFS-like participants were PASC indeterminant with only 8% (377/4692) assigned to cluster 4, 9% (410/4692) assigned to cluster 3, 6% (300/4692) assigned to cluster 2, and 10% (471/4692) assigned to cluster 1 (Fig. 3).

## DISCUSSION

Because ME/CFS can be a consequence of acute infection with viral and non-viral agents,^1,9,10^ we hypothesized it would be a sequela of SARS-CoV-2 infection. The prospective design of the RECOVER-Adult study provided the opportunity to estimate the incidence and prevalence of ME/CFS after infection with SARS-CoV-2. Of the 4515 participants that enrolled in RECOVER Adult within 30 days of their acute SARS-CoV-2 infection and were followed more than 6 months, 2.66 per 100 person-years vs. 0.93 per 100 person-years in matched uninfected participants met the IOM clinical diagnostic criteria for ME/CFS for an attributable risk of ME/CFS after SARS-CoV-2 exposure of approximately 1.74 per 100 person-years. A pre-COVID pandemic study conducted by the CDC estimated the incidence rate of ME/CFS at 0.18 per 100 person-years.^11^ This low incidence rate may reflect the focus on fatigue as the main outcome rather than the full constellation of ME/CFS symptoms. An electronic health record study found the incidence of post-COVID-19 chronic fatigue was 1.8 per 100 person-years.^12^ This is between the rate of ME/CFS we found in the acutely infected and uninfected cohorts and is likely an underestimate because the study relied on ICD coding and, prior to a 2023 update to ICD-10, there was not a specific code for ME/CFS.^13^ Our results provide evidence that the rate and risk of developing ME/CFS following SARS-CoV-2 infection is significantly increased and is supported by other studies that have implicated infectious agents such as Epstein Barr Virus and Ross River Virus and non-viral diseases such as Q fever and giardiasis in the etiology of ME/CFS.^1,9,10^

The 4.5% post-COVID-19 ME/CFS prevalence rate we found in the RECOVER-Adult study is similar to the 3–4% ME/CFS prevalence rate in people with acute infection-like illness suggestive of SARS-CoV-2 infection reported by the Innovative Support for Patients with SARS-CoV-2 Infections Registry (INSPIRE).^14^ However, these post-pandemic prevalence rates are in contrast to pre-pandemic ME/CFS prevalence estimates, which ranged from 0.2 to 1.0% of people in the U.S. and is in line with what we found in uninfected participants.^15–17^ The 2021–2022 wave of the National Health Interview Survey conducted during the pandemic found that 1.3% of adults reported being diagnosed with ME/CFS by a doctor, suggesting that post-COVID ME/CFS might already be increasing national prevalence rates.^18^ Our finding that 4.5% of infected RECOVER-Adult participants met criteria for ME/CFS based on IOM diagnostic criteria is higher than any pre-pandemic prevalence estimate. While this prevalence is confounded by individuals who enrolled up to 18 months after infection and may be differentially less likely to have fully recovered from COVID, when combined with our incidence estimates from those enrolled within 30 days of SARS-CoV2 infection these data provide further evidence of the post-infectious nature of ME/CFS and confirm that it is one of the diagnosable sequelae that occurs after SARS-CoV-2 infection.^19^

Detection and diagnosis of a true positive ME/CFS case is complicated as symptoms vary in frequency and severity over the course and duration of illness.^20–22^ Furthermore, the symptom(s) must be recognized as part of the ME/CFS manifestation. For example, PEM is the worsening of symptoms following physical or cognitive exertion and is an unusual phenomenon, particularly to people who are unfamiliar with the onset, experience, and triggers of exertional intolerance.^23^ PEM is the cardinal feature of ME/CFS. It is the exacerbation of ME/CFS signs and symptoms that can be triggered by daily activities such as showering, driving, reading, cleaning, cooking, or conversing.^23,24^ In this study, PEM was the most common symptom among acute and post-acute infected participants in RECOVER-Adult. PEM has also been identified as one of the most common and debilitating symptoms of long COVID in several studies.^3,25–27^

Cognitive impairment and OI symptoms fluctuate and are exacerbated by being in upright postures.^28–30^ They were the next most common symptoms in RECOVER-Adult study infected participants. While the mechanisms driving these symptoms are not known, there are pathophysiological findings in ME/CFS that help explain these symptoms. Structural, metabolic, and inflammatory abnormalities have been found in the brain of ME/CFS patients.^31–33^ Peripheral neurovascular dysregulation and reduced cerebral blood flow is characteristic of ME/CFS.^34,35^ There is immune dysfunction in both the innate and adaptive immune system in ME/CFS.^36,37^ Before the COVID-19 pandemic, ME/CFS was characterized as a multisystemic metabolic-inflammatory disorder showing altered bioenergetics associated with disease severity, physical exertion, and illness duration.^38–53^

Four PASC subgroups have been identified using cluster analysis in the RECOVER study.^3^ Cluster 1 symptoms include loss or change in smell and taste; cluster 2 includes PEM and fatigue; cluster 3 includes brain fog, PEM, and fatigue; and cluster 4 includes fatigue, PEM, dizziness, brain fog, gastrointestinal, and palpitations.^3^ Cluster 4 is the most symptomatic PASC subgroup with the highest frequency of ME/CFS defining symptoms. This study found that 90% of post-COVID-19 ME/CFS participants met PASC criteria and aligned with the cluster 4 PASC subgroup.^3^ This may indicate that post-COVID-19 ME/CFS represents a severely ill subset of PASC. A careful examination of the pathophysiology in both these groups and, as appropriate, modification, and updating of the ME/CFS diagnostic criteria should be considered.

There are limitations to this study that may have contributed to an overestimation of the number post-COVID-19 ME/CFS participants in RECOVER-Adult. We excluded people with a formal diagnosis of ME/CFS prior to infection but may have missed participants who both had pre-existing qualifying symptoms and were not previously diagnosed, mischaracterizing them as new. There may be recall bias where participants were uncertain whether they had symptoms before or only after infection. We included participants with pre-existing medical and psychiatric conditions that might cause ME/CFS-like symptoms, causing a misattribution of ME/CFS. Severity of PEM and OI were not assessed which might have allowed more participants to qualify. There may be a selection bias since with PASC may be more likely to enroll in RECOVER. These issues are mitigated by the enrollment of participants within 30 days of infection.

Conversely, there are also factors that might have contributed to an underestimation of participants with post-COVID-19 ME/CFS. Hospitalized RECOVER participants were excluded and therefore were not included in the prevalence and incidence estimate of post-COVID-19 ME/CFS. The waxing/waning nature of symptoms might have caused us to miss cases. The 2015 IOM ME/CFS diagnostic criteria mostly reflect severely ill ME/CFS of many years duration and may not be ideal for identifying short duration ME/CFS. Participants might not be familiar with the concept of PEM and may not have reported it. The most severely affected individuals may have been unable to enroll in RECOVER or to have been differentially lost to follow-up because of study burden. Participants were mostly enrolled in the Omicron era, limiting our ability to assess ME/CFS frequency after earlier, more severe variants. Most participants were vaccinated, so incidence and severity may be lower and less than in an unvaccinated population.

ME/CFS is a diagnosable sequela of SARS-CoV-2 infection. RECOVER provides the opportunity to identify objective biomarkers and to study the biology, mechanisms, and natural history of post-COVID-19 ME/CFS.

## Supplementary Information

Below is the link to the electronic supplementary material.

Supplementary file1 (DOCX 15 KB)

Supplementary file2 (DOCX 16 KB)

Supplementary file3 (DOCX 15 KB)

Supplementary file 4 (xlsx 60.3 KB)
