# Supplementary material for: Incidence and Prevalence of Post-COVID-19 Myalgic Encephalomyelitis: A Report from the Observational RECOVER-Adult Study
Source: J Gen Intern Med. 2025 Jan 13;40(5):1085–94. doi: 10.1007/s11606-024-09290-9 (PMC11968624; doi:10.1007/s11606-024-09290-9)
Supplement: Supplementary file 1 — Supplementary file1 (DOCX 15 KB) [file 11606_2024_9290_MOESM1_ESM.docx]

Supplemental Table 1. Demographic and comorbid condition variables used for propensity score matching.

| Demographics |  |
| --- | --- |
|  | Age |
|  | Race |
|  | Sex at birth |
|  | Education level |
|  | Rural residency |
|  | Residency in a medically underserved area |
|  | Pregnancy |
|  | Menopause |
|  | SARS-CoV-2 vaccination status at time of enrollment |
| Comorbid Conditions |  |
|  | Autoimmune disease |
|  | Cancer |
|  | Chronic liver disease |
|  | Sickle cell anemia |
|  | Dementia |
|  | Depression or anxiety disorder |
|  | Bipolar disorder or psychosis |
|  | Chronic pain syndrome or fibromyalgia |
|  | Postural orthostatic tachycardia syndrome |
|  | Neuromuscular disease |
|  | Movement disorder |
