# Supplementary material for: Incidence and Prevalence of Post-COVID-19 Myalgic Encephalomyelitis: A Report from the Observational RECOVER-Adult Study
Source: J Gen Intern Med. 2025 Jan 13;40(5):1085–94. doi: 10.1007/s11606-024-09290-9 (PMC11968624; doi:10.1007/s11606-024-09290-9)
Supplement: Supplementary file 2 — Supplementary file2 (DOCX 16 KB) [file 11606_2024_9290_MOESM2_ESM.docx]

Supplemental Material Table 2. Demographic characteristics of acute infected, uninfected, and propensity score matched uninfected participants of the RECOVER-Adult study.

|  | Demographic Characteristics (%) | | |
| --- | --- | --- | --- |
|  | Acute Infected Group | Uninfected Group | Matched Uninfected Group |
| N | 4515 | 1439 | 4515 |
| Age category at enrollment |  |  |  |
| 18-45 | 44.0 | 45.9 | 43.6 |
| 46-65 | 38.1 | 36.9 | 35.4 |
| >65 | 17.8 | 17.0 | 21.0 |
| Missing (<18) | 0.2 | 0.1 | 0.1 |
| Sex at birth |  |  |  |
| Female | 69.4 | 67.0 | 70.6 |
| Male | 29.9 | 32.5 | 29.2 |
| Missing | 0.7 | 0.5 | 0.2 |
| Race |  |  |  |
| Asian, non-Hispanic | 7.4 | 6.3 | 6.8 |
| Black, non-Hispanic | 12.9 | 19.8 | 18.2 |
| Hispanic | 9.4 | 9.2 | 7.4 |
| Multiracial/ethnic | 7.2 | 7.6 | 7.1 |
| White, non-Hispanic | 60.3 | 54.6 | 58.8 |
| Other | 1.7 | 1.5 | 1.1 |
| Missing | 1.1 | 1.0 | 0.6 |
| Vaccine at enrollment |  |  |  |
| Yes | 76.8 | 91.2 | 75.9 |
| No | 3.0 | 4.5 | 3.4 |
| Don’t know | 0.0 | 0.1 | 0.0 |
| Missing | 5.4 | 0.0 | 0.0 |
| Prefer not to answer | 14.9 | 4.2 | 20.7 |
| Education |  |  |  |
| Bachelors/advanced degree | 72.7 | 62.5 | 75.8 |
| High school/some college | 24.6 | 32.2 | 21.7 |
| Not complete high school | 1.7 | 4.6 | 1.7 |
| Missing | 1.0 | 0.8 | 0.8 |
| Rural |  |  |  |
| No | 97.5 | 95.2 | 98.0 |
| Yes | 2.5 | 4.8 | 2.0 |
| Medically underserved area |  |  |  |
| No | 76.9 | 72.3 | 78.0 |
| Yes | 23.1 | 27.7 | 22.0 |
