# Supplementary material for: Incidence and Prevalence of Post-COVID-19 Myalgic Encephalomyelitis: A Report from the Observational RECOVER-Adult Study
Source: J Gen Intern Med. 2025 Jan 13;40(5):1085–94. doi: 10.1007/s11606-024-09290-9 (PMC11968624; doi:10.1007/s11606-024-09290-9)
Supplement: Supplementary file 3 — Supplementary file3 (DOCX 15 KB) [file 11606_2024_9290_MOESM3_ESM.docx]

Supplemental Material Table 3. Comorbidities of acute infected, uninfected, and propensity score matched uninfected participants of the RECOVER-Adult study.

|  | Comorbidities (%) | | |
| --- | --- | --- | --- |
|  | Acute Infected Group | Uninfected Group | Matched Uninfected Group |
| N | 4515 | 1439 | 4515 |
| Pregnancy | 3.7 | 3.4 | 4.7 |
| Menopause | 19.4 | 24.9 | 20.6 |
| Autoimmune disease | 9.2 | 11.2 | 15.6 |
| Cancer | 2.7 | 4.2 | 2.0 |
| Chronic liver disease | 1.4 | 2.6 | 1.3 |
| Sickle cell anemia | 0.2 | 1.1 | 0.3 |
| Dementia | 1.7 | 2.2 | 1.3 |
| Depression or anxiety disorder | 27.7 | 36.9 | 27.7 |
| Bipolar disorder or psychosis | 2.0 | 5.7 | 1.6 |
| Chronic pain syndrome or fibromyalgia | 4.0 | 7.1 | 4.7 |
| Postural orthostatic tachycardia syndrome | 1.0 | 1.7 | 1.2 |
| Neuromuscular disease | 3.4 | 5.9 | 2.8 |
| Movement disorder | 1.1 | 2.0 | 1.0 |
